# TRIM family proteins: roles in proteostasis and neurodegenerative diseases

**DOI:** 10.1098/rsob.220098

**Published:** 2022-08-10

**Authors:** Yan Zhu, Lukman O. Afolabi, Xiaochun Wan, Joong Sup Shim, Liang Chen

**Affiliations:** ^1^ Shenzhen Laboratory of Tumor Cell Biology, Institute of Biomedicine and Biotechnology, Shenzhen Institute of Advanced Technology, Chinese Academy of Sciences, Shenzhen 518055, People's Republic of China; ^2^ University of Chinese Academy of Sciences, Beijing 100864, People's Republic of China; ^3^ Cancer Centre, Faculty of Health Sciences, University of Macau, Avenida da Universidade, Taipa, Macau, People's Republic of China

**Keywords:** neurodegenerative diseases, trim proteins, protein aggregation, proteostasis, ubiquitin–proteasome system, chaperone

## Abstract

Neurodegenerative diseases (NDs) are a diverse group of disorders characterized by the progressive degeneration of the structure and function of the central or peripheral nervous systems. One of the major features of NDs, such as Alzheimer's disease (AD), Parkinson's disease (PD) and Huntington's disease (HD), is the aggregation of specific misfolded proteins, which induces cellular dysfunction, neuronal death, loss of synaptic connections and eventually brain damage. By far, a great amount of evidence has suggested that TRIM family proteins play crucial roles in the turnover of normal regulatory and misfolded proteins. To maintain cellular protein quality control, cells rely on two major classes of proteostasis: molecular chaperones and the degradative systems, the latter includes the ubiquitin-proteasome system (UPS) and autophagy; and their dysfunction has been established to result in various physiological disorders including NDs. Emerging evidence has shown that TRIM proteins are key players in facilitating the clearance of misfolded protein aggregates associated with neurodegenerative disorders. Understanding the different pathways these TRIM proteins employ during episodes of neurodegenerative disorder represents a promising therapeutic target. In this review, we elucidated and summarized the diverse roles with underlying mechanisms of members of the TRIM family proteins in NDs.

## Graphical abstract

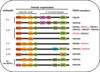

## 1. Introduction

Neurodegenerative diseases (NDs) are a diverse group of disorders characterized by the progressive degeneration of the structure and function of the central or peripheral nervous systems. Aetiologically, many NDs share and are associated with misfolding and aggregation of specific polypeptides and the ensuing loss of neurons [1]. Although some of these diseases are caused by germline mutations that result in the production of defective proteins, the vast majority of these diseases are sporadic and are caused by the aggregation of normal proteins expressed at physiological levels. The most common type of aggregated proteins are amyloid fibril, which is made up of *β* strands that makes up the cross-β structure [2,3].

Some examples of NDs include Alzheimer's disease (AD), Parkinson's disease (PD), Huntington's disease (HD), amyotrophic lateral sclerosis (ALS), spinocerebellar ataxias (SCAs), frontotemporal dementia (FTD), progressive supranuclear palsy (PSP), dementia with Lewy bodies (DLB), multiple system atrophy (MSA), prion diseases/transmissible spongiform encephalopathies (TSEs) and so on [4]. Among these diseases, PD is the most prevalent [5,6]. Interestingly, the process of protein aggregates in NDs differs; for example, AD harbours numerous inclusion bodies containing β-amyloid (A*β*) [7] and tau proteins [8]. Whereas in MSA, PD, and DLB, they are linked with abundant inclusions containing α-synuclein [9,10]; while mutant huntingtin aggregation is found in HD [11].

Since protein misfolding is inevitable and often irreversible, it can result in mutations, biogenetic errors or irreparable damage in the cellular compartment [12]. The structure of proteins determines its functions; and to obtain protein functional three-dimensional structure, and to ensure proper protein folding and avoid proteotoxic stress *in vivo*, cells develop a system known as the protein quality control (PQC) [13]. The PQC is a well-organized, tightly regulated system that includes the molecular chaperone system (ensure the correct folding of proteins) and the degradative pathways: autophagy and the ubiquitin-proteasome system (UPS) (eliminate misfolded proteins once formed) [14]. Molecular chaperones cooperate in de novo folding, refolding of misfolded proteins, maintaining pre-existing native protein structure stability and lowering the level of protein aggregates. When unfolded proteins accumulate, they activate stress responses known as the unfolded protein response (UPR) or the heat-shock response (HSR), depending on the cellular compartment [15]. Interestingly, when misfolded proteins cannot be refolded, the degradative pathways such as the UPS, autophagy and endoplasmic reticulum-associated degradation (ERAD) begin to promote their degradation [16–18]. Thus, the cooperation of these PQC regulating systems is critical in maintaining cellular proteostasis.

Proteostasis disruption has serious consequences such as the pathogenesis of a variety of disorders, including NDs, ageing and cancer [18–20]. Additionally, mutations in genes encoding pathogenic proteins can cause misfolding and sequential aggregation in NDs [21]. The proteostasis network can clear aggregated proteins in the absence of gene mutations; however, this capacity declines with age, resulting in proteostasis dysfunction [22]. Numerous studies have highlighted the neurotoxic effects of misfolded proteins on the disease duration of NDs [23]. Furthermore, chronic expression of misfolded-prone proteins like polyQ impairs the robustness of the proteostasis network, exacerbating misfolding, aggregation, and disease progression and posing a threat to proteostasis integrity [24–26].

**Figure 1.**
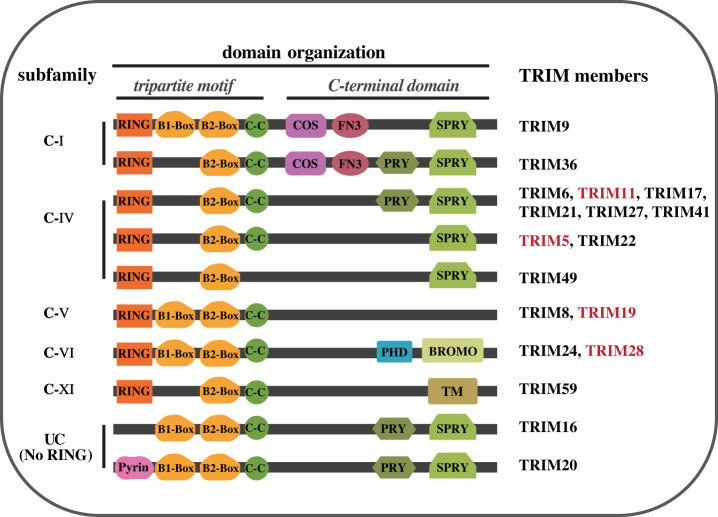
Illustration of TRIM proteins domain structure and classification related to neurodegenerative diseases. Structure classification of TRIM proteins that participate in the pathophysiological processes of neurodegenerative diseases. TRIM proteins share an N-terminal RBCC motif, which consists of a RING domain, followed by one or two B-box domains and a long Coiled-coil (CC) region, some TRIM members lack the RING domain and are classified into the ‘unclassified group’ (UC). Based on their variable C-terminal region, TRIM family proteins are divided into 11 subclasses (C-I to C-XI). C-terminal domains include COS C-terminal subgroup one signature, FN3 fibronectin type 3, PRY-SPRY domain, plant homeodomain (PHD), BROMO bromodomain, TM transmembrane region. This figure illustrates TRIM proteins that play roles in the pathogenesis of neurodegenerative diseases, those TRIM proteins marked in red are elaborated in this review.

The tripartite motif-containing (TRIM) superfamily is structurally defined by TRIM or RBCC motif in its N-terminal region, which contains a RING finger domain, one or two B-boxes, and a coiled-coil domain (CCD) [27,28]. The RING-finger domain contains a Cys3HisCys4 amino acid motif that binds two zinc atoms, and it plays a critical role in ubiquitylation pathways such as recruiting ubiquitin-conjugating enzymes [29,30]. The B box domain can also contain one or two zinc-binding motifs which are typically composed of B1 and B2 domains, but some TRIM members only contain the B2 domain [29,30]. TRIM proteins often self-associate through their coiled-coil regions, forming large protein complexes that later localize in the cytoplasm or nucleus [28]. The CCD is also responsible for interacting with other proteins [29] and it defines the individual function of members of TRIM proteins. TRIM protein family can be classified into sub-family I to XI and one unclassified sub-group that lacks the RING domain but has conserved B-boxes and CCD motif in order and spacing [27,30] (figure 1).

TRIM family proteins play a diverse role in cellular processes including innate immunity [28], genetic disorders [31], cancer [30] and NDs [32]. Our group has shown that several TRIM protein families, including TRIM11/TRIM25/TRIM28, play important roles in regulating cellular proteostasis [32–37]. For example, TRIM11 can facilitate the degradation of defective proteins such as Atxn1 [82Q]—a pathogenic protein that causes spinocerebellar ataxia type1 (SCA1)—in the nucleus and cytoplasm in familial NDs via the proteasome [33,38]. Besides, TRIM11 can reduce USP14 recruitment to the proteasome by inhibiting the association between USP14 and PSMD2, resulting in the promotion of the activity of the proteasome, which further decreases aggresomes and amyloid-like fibrils in cells [34]. We also confirmed that TRIM25 can also promote the degradation of misfolded proteins in the endoplasmic reticulum via ERAD [35].

Other research has found a link between the single nucleotide polymorphism (SNP) rs564309—an intronic variant of the tripartite motif-containing protein 11 (TRIM11) gene—and the clinical phenotype and pathology severity of PSP [39,40]. Thus, emerging evidence suggests that TRIM family proteins play important roles in NDs by regulating proteostasis. This review summarized the roles of TRIM family proteins in regulating protein aggregation and clearance related to NDs via various mechanisms.

## 2. TRIMs functioning as a molecular chaperone or disaggregase

Protein misfolding is the most common pathogenic feature of NDs, manifested primarily by intracellular and/or extracellular protein aggregate deposits [19]. Pathogenic features of NDs include amorphous and fibrillar aggregation [13]; the accumulation of intracellular inclusions containing aggregated α-Synuclein is likely to be a key element in PD [19,41]. Owing to the crowded and complicated intracellular microenvironment, multidomain proteins face significant challenges in achieving native conformation states and maintaining solubility. As a result, the chaperone system acts as a significant buffer for the proper folding of proteins *in vivo* [42].

Molecular chaperones are also known as stress proteins or heat-shock proteins (HSPs) because they are upregulated in response to cellular stress. They are classified according to their size and they include HSP40, HSP60, HSP70, HSP90, HSP100 and other small HSPs [42]. To refold misfolded proteins, molecular chaperones use three distinct approaches, for example, most chaperones, such as HSP70, keep substrate proteins unfolded until the spontaneous fold state is reached [42,43]. In addition, some molecular chaperones, such as HSP70 and HSP60, unfold stable misfolded proteins and restore them to their native protein conformations in an ATP-dependent manner [42,44]. Other chaperones, such as yeast HSP104, are known as ‘disaggregases’ because they dissolve preformed protein deposits and convert them into natively refolded states.

### 2.1. TRIM11

TRIM11, a member of the TRIM family protein that comprises three zinc-binding domains, a RING domain, a type 2 B-box and a coiled-coil region [28]. TRIM11 can prevent protein aggregation and pre-existing protein deposits such as amyloid fibrils in neurodegenerative disorders, thereby relieving the pathogenic features of NDs [13,32–34]. In contrast to canonical chaperones, TRIM11 processed the activity of molecular chaperones by acting in an ATP-independent manner. TRIM11, similar to HSP70 and HSP40, protects model substrates of disease-associated proteins from thermal misfolding, enzymatic inactivation and amorphous aggregation. TRIM11 also functions as a disaggregase, dissolving heat-denatured proteins trapped in amorphous aggregates and restoring their native activities [13].

TRIM11 was shown to be preferentially bound to a pathogenic protein form rather than a non-pathogenic form *in vitro* [13,33], implying that TRIM11 can distinguish misfolded states from the native state of the same protein [13]. TRIM11 abrogated both the de novo and pre-formed fibrils-seed α-Syn fibrillization and prevented the formation of mature fibrils, thereby increasing the soluble α-Syn fraction. Instead of degrading α-Syn proteins, TRIM11 suppresses α-Syn pathology and restores cell models' viability [13]. In the same vein, TRIM11 also inhibited the fibrillization of disease-associated protein, Atxn1 [82Q], the pathogenic protein indicted in SCA1 [12,13,38].

In a mouse model of PD, recombinant TRIM11 injection significantly reduced α-Syn pathology, indicating that TRIM11 does inhibit α-Syn aggregation in mammals. Also, TRIM11 can inhibit dopaminergic neuron loss in the substantial nigra and rescue motor impairments caused by α-Syn aggregation [45]; and animals in the TRIM11 group have better locomotor activity and less anxiety [13].

Based on the evidence presented above, TRIM11 inhibits the seeding and transmission of α-Syn fibrils between cells via its molecular chaperones, and disaggregates activity; it can also rescue mice neurodegeneration and accompanying motor impairments.

### 2.2. TRIM19 and TRIM21

The promyelocytic leukemia protein (PML) is also known as TRIM19. It has the classic TRIM/RBCC motif at the N-terminus and localizes to nuclear structures, but it lacks a recognizable C-terminal domain [46]. TRIM21 is also known as Ro52. It comprises a RING domain, a B-box domain, a coiled-coil domain, and a PRY-SPRY/B30.2 region at its C-terminal [28,47].

TRIM19 and TRIM21, like TRIM11, can degrade Atxn1 [82Q] using their chaperone and disaggregases activities. Notably, TRIM21 can prevent heat inactivation of proteins and solubilize pre-denatured ones; it also can discern misfolded states of proteins [13]. TRIM19, on the other hand, can reactivate thermally misfolded proteins both *in vivo* and *in vitro*. It also has disaggregases activity, which allows it to dissolve Atxn1 [82Q] found in nuclear bodies [13].

In summary, TRIM19 and TRIM21 can act as chaperones and disaggregases in the same way that TRIM11 does in degrading protein aggregations without the need for ATP hydrolysis.

## 3. TRIMs involved in the ubiquitin-proteasome pathway

Ubiquitylation is a post-translational modification that involves the attachment of ubiquitin molecules to a target protein. The ubiquitin-proteasome pathway is crucial for the degradation of most cellular proteins, including short-lived, misfolded proteins and other important proteins related to intracellular processes [48,49].

Ubiquitylation consists of three steps: 1-ubiquitin-activating enzymes (UBA) (E1 enzymes), 2-conjugation by ubiquitin-conjugating enzymes (UBCs) (E2 s) and 3-attachment to the substrate protein by ubiquitin ligases (E3 s). This is done via a multi-step enzymatic cascade, to transfer the ubiquitin moieties to the lysine residues of target proteins [50,51]. The ubiquitinated target proteins are transferred into the core of the proteasome for degradation. The E3 ligases determine the specificity of ubiquitination; a number of these E3 ligases participate in the clearance of toxic aggregate-prone proteins that are linked to NDs [52,53]. Interestingly, TRIM family proteins can function as canonical RING-type E3 ligases and help to clear pathogenic related proteins.

As stated above, certain members of the TRIM protein family are capable of eliminating pathogenic proteins via the ubiquitin-proteasome pathway. Some of these TRIM protein families are discussed below:

### 3.1. TRIM11

TRIM11 processes SUMOylation (SUMO, small ubiquitin-like modifier) activity in addition to chaperone and disaggregase activity to promote the degradation of misfolded proteins. TRIM11 promoted the SUMOylation of Atxn1 [82Q] with the presence or absence of exogenous SUMO2 proteins [12,13]. However, its SUMO ligase activity is dependent on the identity and/or folding state of substrates, as TRIM11 failed to ubiquitinate soluble α-Syn, a normally folded protein. Taken together, it appears that TRIM11's chaperone/disaggregase and SUMO ligase work together to remove abnormal proteins [13].

Our research team has previously demonstrated that TRIM11 inhibited the association of USP14 with PSMD2 and thus reduced the recruitment of USP14 to the proteasome, preventing its association with the USP14 and thus increasing proteasome activity. These findings revealed that TRIM11 increases the proteasome's degradative capacity [34].

We also observed that TRIM11 promotes the degradation of both abnormal and normal regulatory proteins, as well as increases the overall rate of proteolysis. TRIM11 contributes to the degradation of the misfolded proteins, and its effects are more pronounced in a heat shock environment. TRIM11 activated the proteasome and prevented the decline in proteasomal activity under thermally stressed conditions, rather than promoting the ubiquitination of normal regulatory proteins or misfolded proteins—a feature that differed from TRIM11's canonical function [34].

Moreover, TRIM11 has been shown to interact with Atxn1[82Q] and promote its ubiquitination for sequential proteasome degradation. Overexpression of TRIM11 reduced Atxn1[82Q] inclusions as well as Atxn1[82Q] protein levels in cell lysate fractions. Similarly, TRIM11 reduced cytoplasm-localized Httex1p-97QP (a pathogenic protein associated with HD) as it did for Atxn1[82Q] [12,33]. Furthermore, endogenous misfolded proteins such as K48 polyUb-modified proteins and defective ribosome products were also reduced by TRIM11 [33].

Based on the above findings, TRIM11 regulates misfolded protein degradation through multiple pathways, implying a promising role for TRIM11 in the treatment of neurodegenerative disorders.

### 3.2. TRIM19/PML

TRIM19/PML removes a variety of protein aggregates in the nucleus associated with NDs such as HD, ALS, and frontotemporal lobar degeneration with ubiquitinated inclusions (FTLD-U). For instance, Httex1p-97QP inclusions that form in the nucleus can be eliminated by PML, but those that form in the cytoplasm cannot [12,54]. In comparison to non-pathogenic substrates, PML interacts strongly with pathogenic substrates, and following its recognition, PML uses its SUMO ligase activity to conjugate these proteins with SUMOs. Similarly, PML can reduce the abundance of a specific pathogenic ataxin-1 protein (Atxn1 [82Q]) instead of a nonpathogenic ataxin-1 protein (Atxn1 [30Q]) [12].

According to a study conducted by Guo *et al*. the overexpression of RNF4 was shown to reduce the level of aggregated Atxn1 [82Q] in the cell lysates as well as Atxn1 [82Q] inclusions in the nucleus. In addition, RNF4 reduced the half-life of aggregated but not soluble Atxn1 [82Q] proteins. Interestingly, PML and RNF4 formed a mutual dependence relationship in degrading pathogenic misfolded proteins in the mammalian nucleus; RNF4 can also recognize SUMOylated misfolded proteins and then ubiquitinate them for subsequent proteasomal degradation [12]. This research establishes RNF4's critical role in the degradation of misfolded proteins, particularly those implicated in NDs.

Even though PML lacks recognizable C-terminal regions, it is still able to reverse the denatured state of thermally impaired proteins [13]. In the presence of PML, the pathogenic Atxn1 protein was destabilized, and its level decreased by 20%. Furthermore, overexpression of PML would hasten the degradation of aggregated proteins and reduce their half-life, whereas it would not affect the condition of normal Atxn1 protein. As a result, PML can form direct associations with polyQ proteins, preferring the pathogenic form [12].

Together, PML and RNF4, a SUMOylation-dependent ubiquitin ligase, play an important role in protecting against NDs by SUMOylating misfolded proteins and corporately promoting protein degradation.

### 3.3. TRIM8, TRIM22 and TRIM36

The structure of TRIM8 is composed of a RING finger domain, two B-box domains, and a coiled-coil domain at the N-terminal region [31]. According to report, TRIM22 and TRIM36 belong to different subgroups but share the same N-terminal structure, comprising a RING finger domain, one B-box type2 region, and a coiled-coil domain [55]. Based on our previous research, we found that TRIM8, 22 and 36 genes have elevated expression levels during oncogenic transformation, and silencing these TRIM genes impaired the ability of the oncogenic transformed PHMLER (transformed primary human mammary epithelial cells) cells to degrade Atxn1 [82Q], albeit they bolster the steady-state levels of Atxn1 [82Q] in the cells [33].

As a transcription factor, Nrf2 regulates intracellular antioxidant responses and the expression of proteasome subunit proteins [56]. The deregulation of Nrf2 significantly reduced the expression of TRIM8/22/36 in oncogenic transformed PHMLER cells. More importantly, the dysregulation of Nrf2 in PHMLER cells showed the impaired degradative capacity of pathogenic protein aggregates Atxn1 [82Q] and Httex1p-97QP and increased the number of aggresomes, implying that Nrf2 positively regulates the degradation of misfolded proteins partially due to the upregulation of proteasome activity [33]. In summary, TRIM8, TRIM22 and TRIM36 work together to degrade misfolded proteins, possibly via the UPS; however, the precise mechanism of degradation requires further investigations.

### 3.4. TRIM21

AD has two distinct pathogenic protein aggregates: extracellular amyloid-β plaques cleaved from amyloid precursor proteins (APPs) and intracellular neurofibrillary tangles composed of the microtubule-associated protein tau [57,58]. The accumulation of A*β* proteins and hyperphosphorylated tau proteins in neurofibrillary tangles, as well as neuroinflammation, are the most important causes of the development and progression of AD [59,60].

TRIM21 is regarded as an autoantigen, and has been shown to interact with interferon regulatory factors; based on these interactions, it is a crucial regulator of the type I interferon immune response [61]. The cytosolic Fc receptor of TRIM21 is widely expressed and active in a variety of tissues, including the CNS and neural-derived cells. IFNs (IFN-α (type I) and IFN-β (type II)) can stimulate TRIM21 expression, resulting in potentiated neutralization activity [62]. Besides, TRIM21 is also capable of detecting antibody-bound tau proteins and sequentially reducing tau protein degradation [63].

Microtubule-associated protein tau lesions show higher associations with dementia severity than Aβ plaques; thus, targeting pathogenic tau protein is very effective in clinical treatment [64]. McEwan and colleagues established tau ‘seeding’ procedures in human cells (similar to cytoplasmic protein aggregates in Alzheimer patients) and discovered that TRIM21 could intercept and neutralize antibody-labelled tau assemblies. Their study reveals that the intracellular immune system can react to self-propagating misfolded proteins, which has significance for ongoing efforts to create antibody-based treatments for NDs [63]. A recent study found that infusing anti-tau protein antibodies into mouse disease models can drastically reduce tau pathology [65].

It is worth noting that TRIM21 neutralizes tau seeding within the cellular compartment in a way similar to how it defends against viral invasion. TRIM21 is recruited to antibody-labelled tau aggregates upon their entry into the cytoplasm to induce proteasome- and valosin-containing protein (VCP) or p97 (a type of molecular unfoldase involved in multiple cellular functions, including protein degradation via the UPS-dependent tau seeding degradation). VCP, on the other hand, plays a more dominating role in the neutralization process [63]. These findings suggest that a TRIM21-dependent neutralizing response on target pathogenic proteins may represent a promising treatment option for tau-seeding diseases.

## 4. TRIMs involved in autophagy

The autophagy pathway helps to remove soluble cytosolic proteins as well as proteins aggregated into irreversible complexes or aggregates. When the autophagic system is compromised, damaged proteins accumulate in the form of protein inclusions [66]. The autophagy pathway is also responsible for degrading long-lived proteins and damaged organelles via the lysosomes [67]. Autophagy is required to keep the central nervous system (CNS) functioning normally by preventing the accumulation of misfolded and aggregated proteins. Impairment of autophagy has consistently been linked to the pathogenesis of different neurodegenerative disorders [68].

Under normal conditions, brains contain few autophagosome vesicles; however, autophagosome vesicles are found in dystrophic neurites in AD brains. The accumulation of autophagy vacuoles is most likely due to impaired clearance rather than autophagy induction [57]. Autophagy receptors can identify signals on specific cargo proteins such as aggregated proteins, damaged mitochondria, excess peroxisomes, and invading pathogens [69]. Emerging evidence suggests that TRIM proteins play a variety of roles in selective autophagy [70,71]. The following section discusses the involvement of selected TRIM proteins in the clearance of neurodegenerative protein aggregates via the autophagy pathway.

### 4.1. TRIM5*α*

TRIM5*α* includes a RING, B-box and coiled-coil (RBCC) domains [72]. TRIM5*α* was shown to function as a receptor for selective autophagy [70]. According to earlier studies, TRIM5*α* has been implicated with autophagy receptor p62. p62 knockdown inhibited TRIM5*α*-mediated retroviral restriction in cells expressing epitope-tagged TRIM5*α* or endogenously expressed human TRIM5*α*. As a result, p62 may function to augment TRIM5*α*-mediated retroviral restriction, contributing to the antiviral state of cells after IFN treatment [73].

TRIM5*α* colocalized with the ULK1(the Ser/Thr protein kinase, also called Atg1 in yeast) complex and interacted with it; the SPRY domain of TRIM5*α* is required for its binding, indicating that TRIM5*α* has a role in the early stages of the autophagy pathway [70]. TRIM5*α* serves as a platform for assembling and activating ULK1 and Beclin1, thereby initiating autophagy [70].

Importantly, the HIV-1 capsid protein p24 is an autophagy degradation substrate that is dependent on several factors (Atg7, Beclin1, p62, ALFY and TRIM5). TRIM5*α*'s LIR-1 and LIR-2 are vital motifs required for their interaction with mammalian Atg8 s and are also required for autophagy degradation when functioning as an autophagy receptor [70].

These findings show that TRIM5*α* has a non-canonical role in the cytoplasmic quality control pathway that assembles autophagy machinery to aid in the clearance of protein aggregates linked to NDs.

### 4.2. TRIM16

TRIM16 lacks a RING domain at its N-terminus but contains two B-box domains that possess RING-like folds which possessed auto-polyubiquitination activity and act as an E3 ubiquitin ligase, one coiled-coil region and one SPRY domain [74]. In addition, TRIM16 homodimerized with itself and heterodimerized with other members of the TRIM family with the help of the coiled-coil domain [74]. The SPRY domain is known to facilitate protein–protein interactions [75] and is required for p62 and Nrf2 binding. The B-box domains, on the other hand, impede the interaction of p62 with TRIM16 [76].

Jena and colleagues discovered that TRIM16 is essential for protein aggregation assembly, they also observed that TRIM16 functions as a critical regulator in response to oxidative or proteotoxic stress, primarily by stimulating the synthesis and degradation of protein aggregates [76]. An earlier report revealed that upregulation of p62 and p62-containing aggregates caused by deregulation of autophagy are associated with NDs and cancer [77]. The ablation of the gene p62 suppressed the accumulation of ubiquitin-positive protein aggregates in neurons, indicating that p62 is required for the formation of inclusion bodies; however, the deletion of p62 did not influence neuronal degeneration [78].

Importantly, TRIM16 increased the expression of ubiquitin system pathway genes, which are required for the ubiquitination of misfolded proteins that form protein aggregates in a sequential manner. Nrf2 plays a critical role in this process, and its deletion reduced the formation of Ub, p62 and LC3B-marked protein aggregates [76]. TRIM16 serves as a scaffold protein, interacting with p62, ULK1, ATG16L1 and LC3 to promote autophagic degradation of protein aggregates [76]. These studies demonstrate that TRIM16 is physically present on protein aggregates and works in tandem with the autophagy adaptor protein p62, the initiation protein ULK1, and the elongation protein ULK2 (ATG16L1, LC3B). These findings imply that TRIM16 is necessary for the degradation of protein aggregates designated for autophagy.

## 5. TRIMs involved in other proteostasis mechanisms

Recent studies have focused on the roles of TRIM proteins in the proteolysis of defective proteins; however, many TRIM proteins function in the cellular protein quality control system, including during the onset of NDs characterized by protein aggregation formation. Aside from the ubiquitin-proteasome system and autophagy pathways, metazoans have a repertoire of protein quality control systems that function in the coordination, recycling and maintenance of proteostasis. The following section discusses the involvement of selected TRIM proteins in the clearance of neurodegenerative protein aggregates via other mechanisms.

### 5.1. TRIM17 and TRIM41

α-Synuclein (SNCA) is a presynaptic neuronal protein associated with PD both genetically and neuropathologically. SNCA gene is the first gene thought to be mutated in familial PD which encodes α-synuclein [79]. Besides, ZSCAN21 (also known as Zipro1/RU49/ZNF38) is a transcription factor that can target the first intron of the SNCA gene [80] and promote the transcription of this SNCA gene [81,82]. α-Synuclein may contribute to PD pathogenesis in a variety of ways, but it is widely assumed that its aberrant soluble oligomeric conformations, known as protofibrils, are the toxic species that facilitate disruption of cellular homeostasis and neuronal death via its effects on various intracellular targets, including synaptic function [83].

Structurally, both TRIM17 and TRIM41 comprise a RING finger domain, one B-box domain and one coiled-coil domain at its N-terminal, it also possesses a PRY-SPRY domain at the C-terminal region [72,84]. According to a report, ZSCAN21 appears to bind primarily to TRIM17 and TRIM41 among all members of TRIM proteins [81]. Previous data have demonstrated that TRIM17's E3 ubiquitin ligase activity is required and capable of initiating neuronal death by mediating ubiquitination and degradation of Mcl-1 [85,86].

ZSCAN21 can stimulate SNCA transcription in neuronal cells and besides the function of TRIM41 in immune response, catalyzes the ubiquitin-mediated degradation of other substrates including ZSCAN21. Interestingly, TRIM17 can reverse this ubiquitination, stabilizing ZSCAN21 and inhibiting TRIM41-induced ubiquitination [81], further establishing its regulatory role on TRIM41. According to emerging evidence, TRIM17 and TRIM41 prefer homotypic rather than heterotypic interactions, resulting in significantly reduced interaction between ZSCAN21 and TRIM41 in the presence of TRIM17 [81,87].

The study also showed that patients with familial PD have rare genetic variants in the ZSCAN21, TRIM17 and TRIM41 genes. The expression of these variants in the ZSCAN21 and TRIM41 genes causes the ZSCAN21 protein to be stabilized. This study suggests that deregulation of the TRIM17/TRIM41/ZSCAN21 pathway may be involved in the pathogenesis of PD [81], targeting this pathway holds a lot of promise for PD treatment.

### 5.2. TRIM28

Some NDs are caused by the harmful gain-of-function of cellular proteins inside brain neuron cells. Increased levels of α-Syn, for example, have been proven to promote neurotoxicity in PD, while tau protein neuronal aggregation is a hallmark of AD, and their elevated levels including alteration in their subcellular location have been confirmed to induce neurodegeneration in humans and different model species. Despite clinical distinctions, multiple lines of evidence demonstrate that α-Syn and tau proteins overlap pathologically in AD and PD [88].

Structurally, TRIM28 contains a RING domain, two B-boxes, and a coiled-coil region, with intrinsic E3 Ubiquitin ligase activity. TRIM28 has been shown to promote α-syn and tau proteins accumulation in the nucleus via its E3 ubiquitin ligase activity, resulting in the pathogenesis of PD or AD [88].TRIM28 regulates the pathogenic stage of α-syn and tau proteins post-translationally, and any slight decrease in TRIM28 abundance can lead to a decrease in both α-syn and tau protein aggregates. Using transgenic Drosophila tauopathy model and α-syn-overexpression-induced Parkinsonism mouse model confirmed that suppressing TRIM28 reduced the accumulation of pathogenic proteins α-syn and tau proteins in the nervous system [88].

TRIM28 was found to negatively regulate the SUMOylation of both α-Syn and tau proteins. Since SUMOylation has earlier been proposed to facilitate the nuclear localization of certain proteins, it was postulated that TRIM28 may act as a SUMO ligase for α-Syn and tau proteins [89]. While this assertion may be true, more research should be undertaken to uncover such a mechanism using genetically engineered mice that allow monitoring such modification *in vivo*.

Taken together, these findings revealed that TRIM28 drives the nuclear accumulation of α-Syn and tau proteins, implying that inhibiting TRIM28 late in the pathogenic process of these protein aggregates may hold promising therapeutic benefits with fewer side effects.

## 6. Discussion and future perspectives

**Figure 2.**
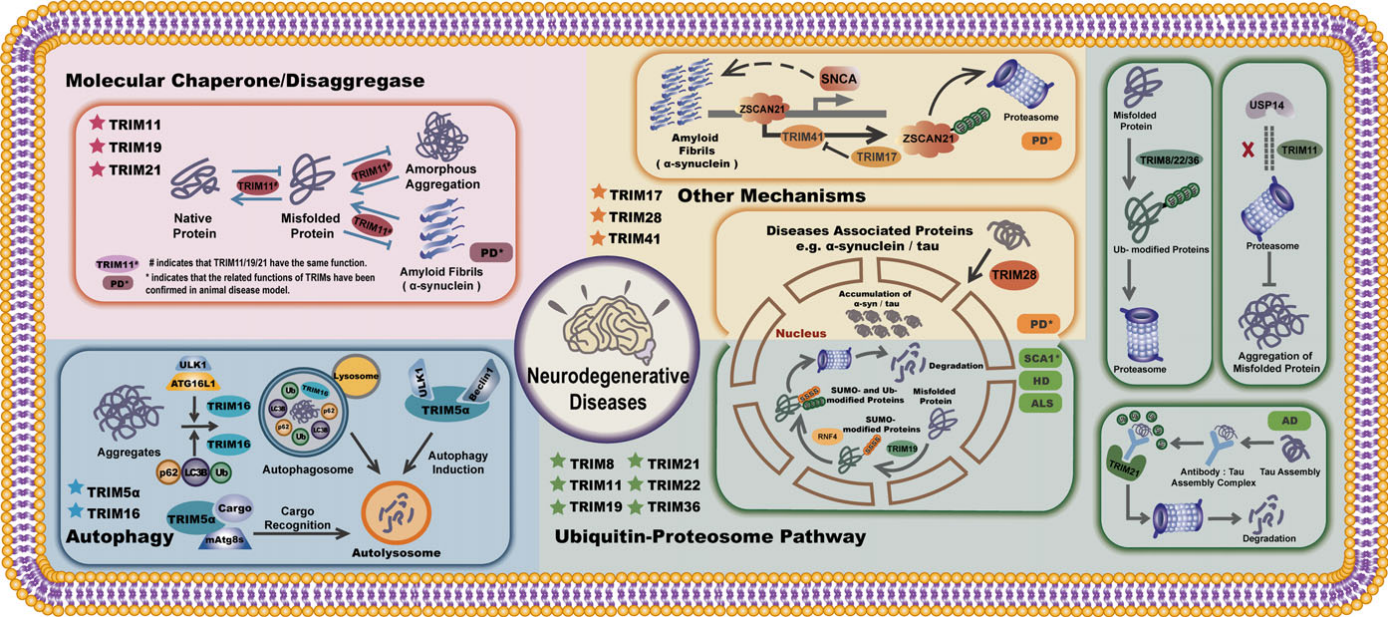
Molecular mechanisms of TRIM proteins in clearing misfolded proteins and protein aggregate via different mechanisms. Molecular chaperone/disaggregases: TRIM11 functions as a chaperone and a disaggregase, preventing the formation of protein aggregates and dissolving pre-existing protein deposits in an ATP-independent manner. TRIM11 also augments the native protein folding and solubility; the chaperone and disaggregases activity of TRIM11 has been confirmed in the Parkinson's diseases mouse model. TRIM19 and TRIM21 function similarly to TRIM11 in preventing and reversing protein aggregation. Ubiquitin–proteasome pathway: (1) TRIM19 promotes the SUMOylation of misfolded proteins. SUMOylated proteins are further ubiquitinated by RNF4 and are subsequently degraded by UPS. The role of TRIM19 in protecting against NDs has been confirmed in a mouse model of SCA1. TRIM11 inhibits the association of USP14 with PSMD2 and reduces its recruitment to the proteasome. TRIM11 specifically inhibits USP14's de-ubiquitinase activity, whereas forced USP14 expression significantly increased aggresomes and amyloid-like fibrils in cells. (2) TRIM8/22/36 promotes the ubiquitination of misfolded proteins via UPS. (3) The cytosolic Fc receptor of TRIM21 can detect antibody-bound tau proteins and degrade the tau assembly complexes via UPS and VCP (a type of molecular unfoldase). Autophagy: (1) TRIM5*α* serves as a scaffold for the assembly and activation of both ULK1 and Beclin1, thereby initiating autophagy. TRIM5*α* also acts as a selective autophagy receptor, delivering cargo protein for autophagy degradation via interacting with mammalian Atg8. (2) TRIM16 acts as a scaffold protein and interacts with p62, ULK1, ATG16L1 and LC3 to facilitate autophagic degradation of protein aggregates. TRIM16 also leads to the ubiquitination of misfolded proteins and the clearance of protein aggregates. Other mechanisms: (1) ZSCAN21 stimulates the transcription of SNCA (gene encoding α-synuclein). TRIM41 promotes the ubiquitination of ZSCAN21 for subsequent degradation via UPS. TRIM17 can inhibit TRIM41 to stabilize ZSCAN21 thus allowing ZSCAN21 to be stabilized and thus favouring α-syn expression. A mouse model of Parkinson's disease has confirmed this regulating pathway. (2) TRIM28 promotes the nucleus accumulation of α-Syn and tau proteins, leading to the late pathogenic process of PD or AD. A transgenic Drosophila tau disease model, a PD mouse model, and pre-symptomatic mouse models of α-Syn and tau-disorders all showed the involvement of TRIM28 in the pathogenicity of protein aggregates associated with NDs.

In this review, we summarize the emerging roles of the tripartite motif-containing protein family in clearing and regulating protein aggregation associated with the pathogenic process of NDs via the ubiquitin-proteasome pathway, autophagy, and other mechanisms (figure 2). For instance, TRIM21 can detect antibody-bound tau proteins via its cytosolic Fc receptor and further degrade the tau aggregation through UPS and VCP, which provides an opportunity for antibody-based therapy against NDs, especially AD [63]. Several pieces of research show promising results from animal model experiments in treating PD; TRIM11, for example, can prevent and reverse protein aggregation through its chaperone and disaggregase activity [13]. In the nucleus, TRIM28 promotes the accumulation of α-Syn and tau proteins, leading to the pathogenic progression of PD or AD [88]. The PML/TRIM19 and RNF4 axis is another regulatory pathway implicated in the nucleus and confirmed in the SCA1 animal disease model, and PML can selectively SUMOylates misfolded proteins. SUMOylated misfolded proteins are sequentially ubiquitinated by RNF4 and finally degraded via the proteasome [12]. Furthermore, silencing ZSCAN21 and TRIM17 can reduce SNCA expression while silencing TRIM41 increases it, confirming that ZSCAN21, TRIM17 and TRIM41 all work together to regulate α-Syn expression in PD [81]. TRIM5 and TRIM16 also function as scaffold proteins, facilitating protein degradation via autophagy, which may be linked to aggregation-related diseases like neurodegeneration [70,76].

A significant number of neurodegenerative illnesses are triggered by the aggregation of normal proteins produced at physiological levels and can develop spontaneously (such as aggregates of β- amyloid fibril) [2,3]. Many recent studies have focused on the emerging roles of TRIM proteins in the clearance of misfolded and protein aggregates. Although members of the TRIM family proteins can be found in different parts of the cell, such as the nucleus, cytoplasm or ER, they perform their functions by facilitating the degradation of misfolded and aggregated proteins via various pathways such as the UPS, autophagy, and other mechanisms.

Apart from the TRIM family proteins mentioned above, a genome-wide association study found that SNPs at the TRIM10, TRIM15 and TRIM26 genes are linked to vulnerability to multiple sclerosis (MS)—a chronic and demyelinating disorder of the central nervous system that has become a major cause of neurological disability in young adults [90–92].

Our previous research also confirmed that the capacity to degrade misfolded proteins by proteasome and autophagy pathway was increased albeit with smaller inclusions formed during HMEC transformation in breast cancer cell lines. Alteration in key genetic elements (including TRIM proteins) in tumour cells has previously been reported to regulate certain physiological processes involved in the cell cycle, cell proliferation, and many signalling pathways including protein turnover [93]. Tumour cells have an enhanced ability to degrade misfolded proteins [94,95], whereas abnormal accumulation of these misfolded proteins (such as Atxn1 [82Q] or Httex1p-97Q) prevented immortalized cell lines from transforming into oncogenic transformed cell lines [33]. Moreover, during anchorage-independent growth, the accumulation of misfolded proteins stimulates a higher level of oxidative stress and thus impairs tumour growth. Meanwhile, tumour cells have an enhanced capacity to degrade misfolded proteins to facilitate malignant growth [33], because misfolded protein accumulation forms protein aggregates that induce pathogenesis of NDs; this suggests another intriguing causal linkage between cancer and onset of neurodegenerative disorders [96–99].

Several emerging therapeutic strategies in treating NDs include regulating and augmenting chaperone levels, adaptively increasing proteasome activity, directly decreasing misfolded protein levels, selectively inactivating the disease-associated mutant allele, and immune approaches are some of the currently available approaches. However, challenges in the delivery system in crossing blood–brain barriers, as well as side effects associated with chemical treatment and immunization approaches, make interventions in treating neurodegenerative disorders difficult to achieve [58]. Therefore, the use of TRIM proteins to regulate protein degradation and clearance in the treatment of NDs and cancer holds a lot of promise.

In the current review, we discovered that several other TRIM proteins do not directly participate in the clearance of misfolded proteins related to NDs, but they have been implicated in playing critical roles in the pathophysiology of NDs other than protein degradation, as well as other functions related to them (figures 1 and 2). For example, TRIM9, a specific brain E3 ubiquitin ligase, participates in the regulation of neuronal functions and the pathogenic progression of NDs through its ligase activity [100]. Also, TRIM59 hypermethylation is linked to cell cycle and DNA repair regulation, which may contribute to AD pathology [101]. Furthermore, the M694 V mutation in the TRIM20/pyrin gene has been shown to contribute to the age at which AD manifests [102]. Apart from TRIM5, many other TRIM proteins, such as TRIM6/17/22/49, also regulate autophagy initiation, acting as a platform for recruiting ULK1 and Beclin1 [70]. According to an interesting study, TRIM27 deficiency can reduce apoptosis and dopaminergic neuron loss, making it a potential target for treating PD [103]. Furthermore, during the neuronal differentiation process, the transcription levels of TRIM6 and TRIM24 were different between PD patients and normal people, which reflects their possible roles in the neurodegeneration pathology process at the early stages of PD [104].

Although we only discussed the identified members of the TRIM protein family implicated in the pathophysiology of NDs in this review, and we highlighted the possible molecular mechanisms by which they are involved in these debilitating diseases, there may be cross-talk between these pathways (UPS, autophagy and other pathways). Since TRIM proteins can form heterodimers with other TRIM proteins, it is critical to investigate the involvement of other unidentified TRIM protein family members to see if they can play cooperative, synergistic, or antagonistic roles during episodes of neurodegenerative pathophysiology.

Misfolded proteins have a proclivity to undergo sequential ubiquitination and SUMOylation mediated by one, two or more TRIM protein members, prompting the direct association between ubiquitination and SUMOylation and how they affect misfolded protein degradation, which will shed more light on the unique roles of these TRIM proteins and require further study [105]. Therefore, it is essential to investigate the crosstalk among these TRIM protein families, particularly how they participate in the degradation of misfolded proteins associated with NDs via the UPS, autophagy, other pathways, or a combination of all.

## Data Availability

This article has no additional data.
